# Process evaluation for enhanced production of aflatoxin-free citric acid by food-grade *Aspergillus niger*

**DOI:** 10.1371/journal.pone.0321972

**Published:** 2025-05-19

**Authors:** Ambreen Latif, Hazrat Ali, Muhammad Bilal Khan Niazi, Zaib Jahan, Noor Hassan, Iqra Latif Ghuman, Abdul Tawab, Anam Saqib, Muhammad Hamid Rashid

**Affiliations:** 1 National Institute for Biotechnology and Genetic Engineering-College (NIBGE-C), Pakistan Institute of Engineering and Applied Sciences (PIEAS), Faisalabad, Pakistan; 2 School of Chemical & Materials Engineering (SCME), National University for Science and Technology (NUST), Islamabad, Pakistan; 3 Department of Chemical Engineering, King Fahd University of Petroleum and Minerals (KFUPM), Dhahran, Saudi Arabia; 4 Interdisciplinary Research Center for Refining & Advanced Chemicals, King Fahd University of Petroleum and Minerals (KFUPM), Dhahran, Saudi Arabia; 5 QuinTech Center for Applied Sciences (QCAS), Lahore, Pakistan; Universidad Autonoma de Chihuahua, MEXICO

## Abstract

*Aspergillus niger* is considered as the main horsepower for the production of 90% of citric acid worldwide. Citric acid is produced under submerged fermentation and the process yield depends on *A. niger* and the process parameters. The current research work was aimed to evaluate the most favorable process parameters for producing the highest amount of citric acid using *A. niger*. The process parameters such as sucrose concentration, pH, temperature, and inoculum size were evaluated for determining their impact on citric acid production. High-performance liquid chromatography (HPLC) analyses revealed different optimal conditions for citric acid production. The optimal conditions found for higher yield of citric acid by *A. niger* included sucrose concentration 12.0% (w/v), temperature 30 °C, pH 5.0 and an inoculum size of 5.0% (v/v). At these optimal conditions, the resulting concentration was 59.0 g/l of citric acid, 24.0 g/l of biomass production, and 2.0 g/l of residual sugar. In addition, food-grade nature of *A. niger* was determined by investigating aflatoxin in production media using HPLC. Our results showed that there were no traces of aflatoxins in the production media, hence the used *A. niger* was of food-grade nature. These findings underline the importance of process evaluation for maximizing citric acid yield and efficiency of *A. niger* for bioprocess engineering and industrial applications.

## 1. Introduction

Microorganisms are being well-known workforce for the synthesis of antibiotics, vaccines, enzymes and many other products. Emerging trends in understanding microbial biotechnology make microorganisms major contributors toward the production of many industrial products and catalysts through fermentation [1]. A thousand years ago, microorganisms played an important role in producing different types of products such as wine, beer, and bread. The comprehension and control of fermentation processes, notably during World War I, resulted in the production of organic acids, e.g., gluconic acid, fumaric acid, lactic acid and citric acid by fermentation process. Meanwhile, citric acid production technique was discovered and became the most widely producing organic acid with multiple applications [2].

Citric acid is known as a weak organic acid having formula C_6_H_8_O_7_, a tricarboxylic acid with a molecular weight of 210.14 Da, which act as an intermediate product in the citric acid cycle. The low toxicity nature makes citric acid one of the highly demanding product for consumption on global level [3,4]. It is a natural preservative and widely used as a versatile organic compound used in food and beverages, and pharmaceutical industry [5]. Citric acid is produced in bulk quantities and ranks as the next most abundant fermentation product following ethanol. The applications of citric acid start from being used in biodegradable packaging components, fruit preservation, disinfectant materials, and extracting agent [6,7] to plays a role in formulating numerous foods as an antioxidant, accidulant, preservative, and emulsifier [8, 9, 10]. Citric acid used in food and pharmaceutical industries should be free of aflatoxin. Aflatoxins (AFs) are a group of immunosuppressive mycotoxins and carcinogenic in nature that are proved harmful to food safety worldwide [11, 12, 13].

Citric acid is being produced either by submerged or solid state fermentation at industrial level, mostly mediated by *A. niger*. To date, citric acid production under submerged fermentation by *A. niger* appears the most desirable way of microbial based production of citric acid. *A. niger* is a filamentous fungus, widely used in various industrial fermentation processes for producing diverse primary and secondary metabolites, e.g., enzymes, proteins, and organic acids, more notably citric acid. However, the main concern with using *A.* species for citric acid production is their ability to produce aflatoxin. These species produce aflatoxin as a secondary metabolite, which are known as immunosuppressive and carcinogenic in nature. Among different types of aflatoxins, AFG_1_, AFG_2_, AFB_1_, and AFB_2_ are the most commonly found and life-threatening types of aflatoxin [14]. Therefore, industries prefer to use *Aspergillus* species for citric acid production with no ability to produce aflatoxins.

However, evaluated fermentation parameters and suitable microbial source, e.g., *A. niger*, are required for economical and successful production of citric acid on industrial scale. Especially, fermentation factors majorly impact citric acid production. These factors include type of substrates, substrate initial concentration, temperature, initial pH, media composition, nutrient concentration, and fermentation time. Hence, the current research work is focused on evaluating different process parameters for developing a process that exert positive impacts on the rate of citric acid production during fermentation process. The citric acid fermentation outlooks as a major process in the field of biotechnological industries [14]. In addition, the current study also focused on exploring food grade *A. niger* with no ability to produce aflatoxins for enhanced production of citric acid.

## 2. Materials and methods

### 2.1. Chemical and reagents

All microscopes used in this research were purchased from Sigma-Aldrich of analytical grade. All solvents used for HPLC analysis were HPLC grade of Merck Company.

### 2.2. Microorganism and culture maintenance

In the current study, the already stored lyophilized spores of *A. niger* were used for citric acid production. The lyophilized spores were obtained from indigenous culture collection bank at National Institute for Biotechnology and Genetic Engineering (NIBGE), Faisalabad, Pakistan. The suspension was prepared by cultivating *A. niger* on potato dextrose agar (PDA) and incubated at 30 °C. After 5–7 d, colonies appeared on the surface of incubated plates. They were added in sterile distilled water and were counted using the Neubauer counting chamber (Sigma-Aldrich^®^). The fungal suspension of conidia was diluted to a concentration of 1 × 10^7^ spores/ml.

### 2.3. Morphological characteristics

*A. niger* was cultured on different media such as PDA (g/l; potato infusion 4, dextrose 20; agar 15), Sabouraud dextrose agar (SDA) (g/l; mycological peptone 10, dextrose 40, agar 15) and Czapek-Dox agar (CDA) (g/l; sucrose 40, dipotassium phosphate 1, sodium nitrate 2, potassium chloride 0.5, magnesium sulfate 0.5, ferrous sulfate 0.01, and agar 15) for 72 h and growth pattern was observed on each media. During the 7 d’ incubation period, the fungal colonies were observed regarding textures, color, and shapes.

### 2.4. Citric acid production tendency

During the primary screening, *A. niger* strain was evaluated for citric acid production. The CDA was used as growth medium with bromocresol purple as an indicator. The plates were incubated for 3 d for development of a yellow zone which indicated citric acid production [15]. The clearing zone index (CI) was calculated by following formula [16];


$$Clearing\;Zone\;Index\;(CI)=\frac{Colony\;diameter+Halo\;zone\;diameter}{Colony\;diameter}$$

### 2.5. Inoculum preparation

Spore suspension of *A. niger* was cultured on autoclaved rice for five days at 30°C. To prevent contamination, the culture is subcultured monthly for reducing contamination and providing fresh nutrients [17]. To create a spore suspension of *A. niger*, sterile distilled water was used to collect spores. Spores were quantified using a Neubauer chamber (Marienfeld German Company) under microscope (ZEISS) and spore density was maintained at 1 × 10^7^/ml.

### 2.6. Production media and fermentation

Two different media were used as production media for citric acid production, named as ‘Media 1’ (g/l); Sucrose, 120.0; KH_2_PO_4_, 1.0; NaNO_3_, 4.0; MgSO_4_.7H_2_O, 0.23; ZnSO_4_, 0.0012; FeCl_3_, 0.02; MnCl_2_·H_2_O, 0.0012 (pH 4 ± 0.3) [18] and ‘Media 2’ (g/l); Sucrose, 120.0; KH_2_PO_4_, 1.0; NH_4_NO_3_, 5.0; MgSO_4_·7H_2_O, 5.0; CuSO_4_.5H_2_O, 0.06; ZnSO_4_, 0.1; FeCl_3_, 0.037; (pH 4 ± 0.3) [19]. Using these media, *A. niger* spores were inoculated in 300 ml culture medium prepared in 1000 ml Erlenmeyer flasks. Every flask was cultured with 2.5% (v/v) inoculum of *A. niger* containing 1 × 10^7^/ml spores. Each flask was subsequently placed at 30 ± 2°C with agitation at 180 rpm for a duration of 8 d in an incubator shaker (ZHWY-211B).

## 3. Evaluation of process parameters

### 3.1. Comparison of citric acid production media, nitrogen source, and trace elements

Two production media, ‘Media 1’ and ‘Media 2’ were prepared and incubated at 30°C for 10 d. The amount of citric acid produced was measured after every 24 h. The yield of citric acid production was assessed using ammonium nitrate and sodium nitrate as ‘Media 1’ and ‘Media 2’ contained different nitrogen sources. In ‘Media 1’, sodium nitrate was used as a nitrogen source, whereas in ‘Media 2’ this source was ammonium nitrate. Citric acid production was also calculated across different concentrations of ammonium nitrate. Later, different experiments were performed to investigate citric acid production against various concentrations of ammonium nitrate ranging from low to high value (3.5 g/l to 5.0 g/l). The effect of trace elements as Cu and Zn on the production of citric acid was investigated in comparison to their absence

### 3.2. Inoculum size and age of spores

Various important factors were considered while evaluating inoculum size, e.g., age, purity source, and size of spores to prepare healthy and robust inoculum for fermentation process. In this experiment, flask was inoculated with various inoculum sizes (v/v) like 1%, 2.5%, 5%, 7.5%, and 10%, and incubated at 30°C for 8 d. The next experiment was performed against the age of *A. niger* spores. In this experiment, suspension of *A. niger* spores was prepared from 3, 5, 7, and 9 d of *A. niger* cultured on rice pieces. Growth media flasks were inoculated from previously described spore’s suspension respectively and incubated at 30°C for 8 d.

### 3.3. Effect of temperature, pH, and incubation period

The impact of temperature during fermentation to produce the highest amount of citric acid was studied at different temperatures 25, 30, 35, and 40°C. For this experiment, different batches of growth media were prepared in 1 L flasks and autoclaved at 121°C for 15 min and inoculated with 5.0%(v/v) *A. niger* spores. In addition, the impact of pH on the fermentation process was also studied. Initial pH of fermentation media was set at different values 3.0, 4.0, 5.0, and 6.0. In this experiment, production media flasks were inoculated with 5.0% (v/v) inoculum, and incubated at 30°C and 180rpm for 8 d.

Furthermore, the citric acid production was evaluated for influence on incubation time. Briefly, the *A. niger* was separately cultivated in the production media using 5.0% (v/v) spores. The production media was incubated at 30°C for 8 d. For all experiments as mentioned above, the sample was taken out after regular intervals of 24 h and analyzed using an analytical technique (HPLC chromatograph).

## 4. Recovery of citric acid from fermentation broth

Once the final production of citric acid was achieved, fermentation broth was heated up to 70°C for 15 min. Later on, broth was filtered to remove mycelia of *A. niger*. Calcium hydroxide was added into filtrate to produce calcium citrate, adjusting pH to 5.8 [20,21]. The resulting calcium citrate was precipitated and removed by filter paper. It is further diluted by the addition of water. Further treated with conc. H_2_SO_4_ to form gypsum and citric acid. Then, gypsum was precipitated and removed by filter paper, leaving a filtrate containing pure citric acid. After that pure citric acid was placed at a temperature of 60–70°C to evaporate water, leading to the formation of pure citric acid crystals [22, 23, 24].

## 5. Quantification of citric acid by HPLC

### 5.1. Sample preparation

First, broth containing citric acid was subjected to centrifugation (10,000 rpm for 15 min). After centrifugation (Beckman Coulter, J2-HS Centrifuge) of fermented broth, the sample was filtered to remove any remaining particulate matter or debris. The sample was diluted with deionized water to bring it within the linear range of the detector. Diluted sample was further filtered through a 0.2 μm syringe filter (PTFE).

### 5.2. Mobile phase preparation

A mobile phase for HPLC was prepared by adding 6.82 g of K_2_HPO_4_ (Fisher Company) in deionized water to make a 10 mM K_2_HPO_4_ phosphate buffer. The buffer pH was adjusted to 3.0 by carefully adding drop-wise concentrated phosphoric acid. The prepared solution was then diluted to a final volume of 1000 mL adding deionized water. To achieve an HPLC-grade mobile phase, solution was filtered using a 0.22 µm nylon membrane under vacuum filtration [21]. The acetonitrile (HPLC-grade) was used to wash the column before and after the performance of experiment to avoid phase collapse.

### 5.3. Standard preparation and HPLC analytical procedure

Citric acid quantification was done through Perkin Elmer HPLC. Analytical grade granular citric acid (Fisher) was used to make citric acid standards with concentrations of 1200, 1400, 1600, 1800, and 2000 ppm by adding it to deionized water. Later on, these prepared standards were transferred into autosampler vials to proceed with Perkin Elmer HPLC analysis. The Perkin Elmer HPLC was equipped with a C18 column (150 mm × 4.6 mm, 5 μ Hypersil; Thermo Fisher Scientific, USA), UV detector (Table 1) [21]. The stationary phase for HPLC was 80% acetonitrile.

**Table 1:** HPLC operational parameters.

Parameter	Value
Column	HPX-87H
Mobile Phase	K_2_HPO_4_ (10 mM) pH 3.0
Mode	Isocratic
Analysis time	25 min
Temperature	25°C
Injection Volume	10 µ L
Wavelength	210 nm

### 5.4. Determining food grade nature of Aspergillus nigera

A 300 mL of cell free supernatant, produced by *A. niger* was extracted using 300 mL of chloroform with the help of separating funnel. Later, chloroform (organic solvent) was evaporated using a rotary evaporator (REV-1000AX, Infitek). The residue was dissolved in 3 mL methanol, filtered through a 0.45 μm, pore size Millipore nylon membrane filter, and used for analysis using UHPLC. During UHPLC analysis, a 10 μL mixture of aflatoxins (aflatoxin G1, aflatoxin G2, aflatoxin B1, and aflatoxin B2,) standards, each at a concentration of 10 ng/mL in acetonitrile, was injected into Ultimate dionex 3000 UHPLC equipped with fluorescent detector (UHPLC-FLD), operated with chromeleon software. This analysis was conducted at an isocratic flow rate of 0.4 mL/min for total 20 min, and a column temperature was kept at 30°C. The mobile phase comprising methanol: acetonitrile: water (22:22:56 v/v) was applied for the whole run.

### 5.5. Statistical analysis

All experiments were done in triplicates. The outcomes from this research were gone through statistical analysis, consisting of Mean and Standard Deviation (SD). All statistical analyses were done using Microsoft Office Excel.

## 6. Results and discussion

### 6.1. Morphological characteristics

**Fig 1:**
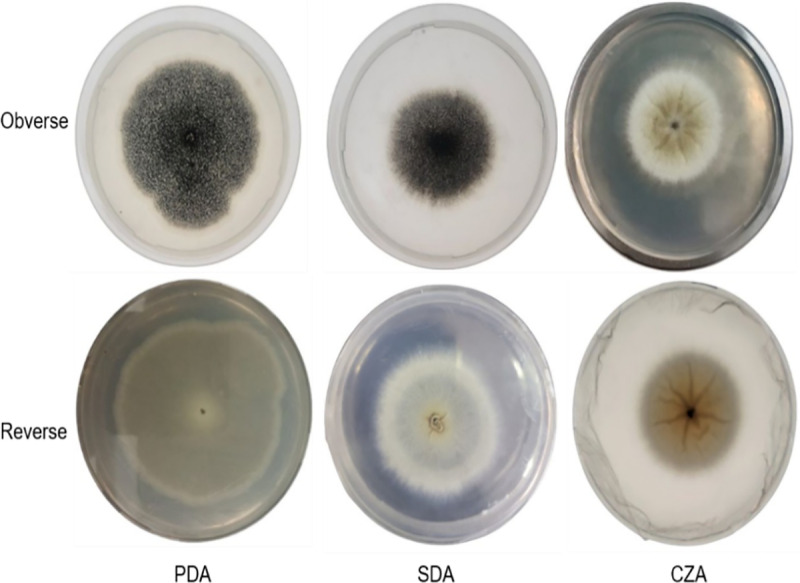
Morphological representation of A. niger growth on different media.

During the morphological observations, the rapid growth was examined mainly on potato dextrose agar medium, while slow growth was observed on Czapek’s media (Fig 1). Similar findings were also observed by Ejimofor [25].

### 6.2. Citric acid production tendency

**Fig 2:**
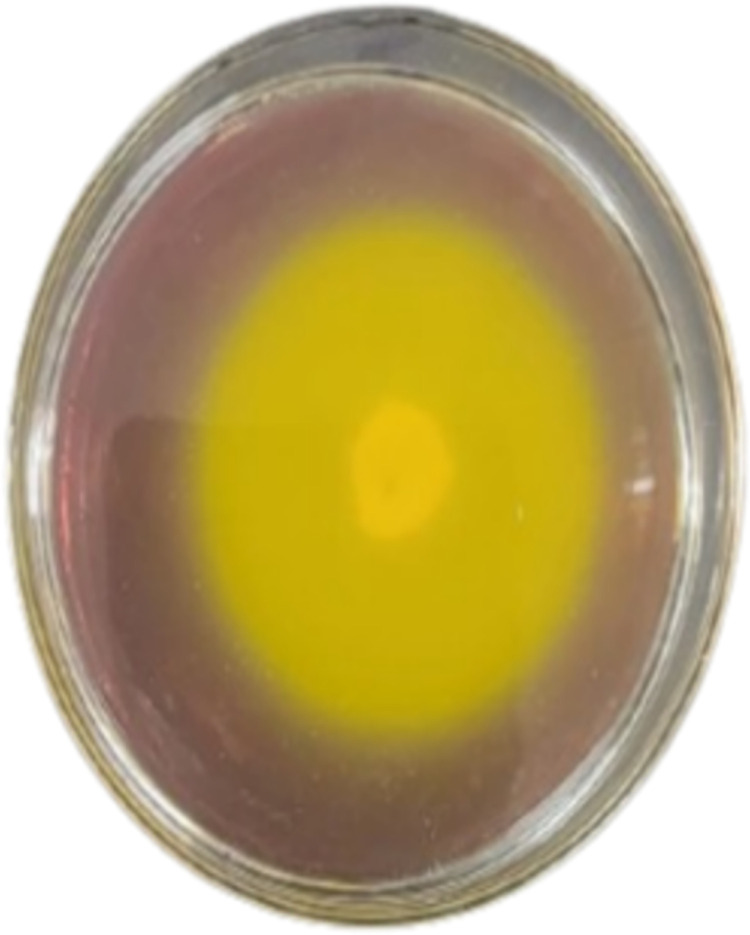
Citric acid-producing zone.

Initially, tendency of existing *A. niger* strain was assessed for citric acid production, and found that the strain was sufficiently producing citric acid (Fig 2). The cleared zone index was achieved 3.5 following a 72 h incubation period at 30°C. Alhadithy and Yasin also reported the same method to find the production tendency of citric acid [26].

**Fig 3:**
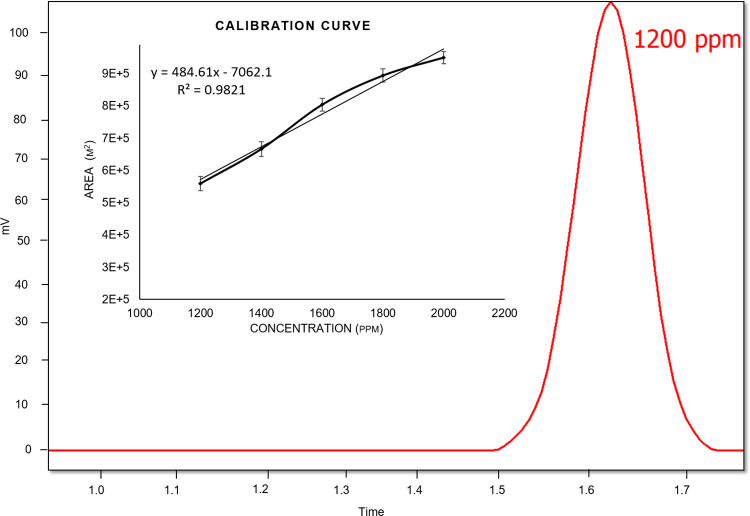
HPLC chromatograph and calibration curve.

In addition, citric acid was quantified using HPLC. The chromatograph presented mV values of 105, 118, 143, 151, and 165 for different concentrations of 1200, 1400, 1600, 1800, and 2000ppm, respectively. For quantification purposes, the area versus concentration (calibration curve) was determined as shown in Fig 3.

### 6.3. Effect of different production media, nitrogen source, and trace elements

**Fig 4:**
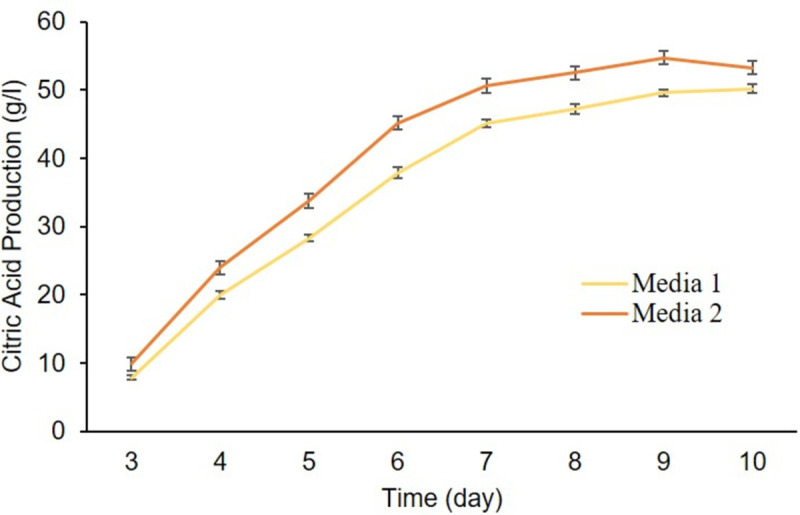
Effect of different production media on citric acid production.

**Fig 5:**
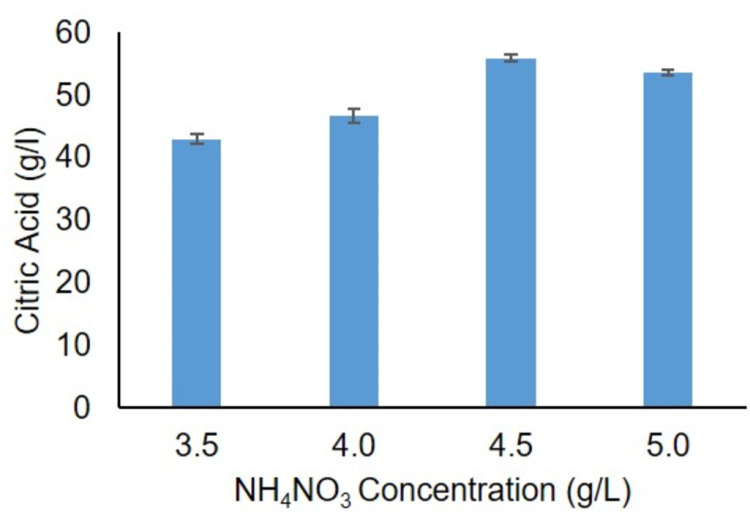
Effect of NH_4_NO_3_ concentration on citric acid production.

Different amount of citric acid was achieved in both ‘Media 1’ and ‘Media 2’. The highest quantity of citric acid (55.0 g/l) was achieved in ‘Media 2’ (Fig 4), and was selected for further studies. Subsequently, different quantity of citric acid concentration was achieved in the presence of various concentrations of ammonium nitrate (3.5–5.0 g/l). The highest citric acid concentration 56.0 g/l was achieved with 4.5 g/l of ammonium nitrate and the lowest citric acid quantity 43.0 g/l was quantified at 3.5 g/l ammonium nitrate concentration (Fig 5). Ammonium nitrate serves as the best nitrogen sources for citric acid production [27]. Our findings are also in agreement with Kareem *et al.* research findings as they reported 60.0 g/kg [28]. Citric acid production starts when nitrogen level drops below a certain threshold. It shows that citric acid is mainly produced by cells that store carbon [29]. Importantly, ammonium nitrate is preferred as it reduces the pH without generating unwanted oxalic acid and it is utilized in the medium [30].

Later on, the highest quantity of citric acid (56.0) g/l was achieved in ‘Media 2’ containing trace elements. Without trace elements, a very low quantity of citric acid (35.0 g/l) was quantified. The presence of trace elements (iron, manganese, iron, zinc copper, and zinc levels) has a considerable influence on the yield of citric acid production. It was observed that when trace elements are at optimal levels, other factors have a less prominent impact [29]. A citric acid concentration of 42.0 g/l was observed in the absence of trace elements. Dienye *et al*., [24] similarly reported a low yield of 12.0 g/l without the addition of trace elements. Upon the addition of trace elements, they achieved a citric acid concentration of 19.0 g/l. Trace element, e.g., Mn^2+^ or Mg^2+^, are important for enzymes such as citrate synthase, isocitrate dehydrogenase, and alpha-ketoglutarate dehydrogenase which governed citric acid production in *A. niger* [31, 32, 33]. Roosterman & Cottrell demonstrated that *A. niger* fungus only maintained growth at high zinc concentrations without citric acid accumulation [33].

### 6.4. Effect of inoculum size and age of spores

**Fig 6:**
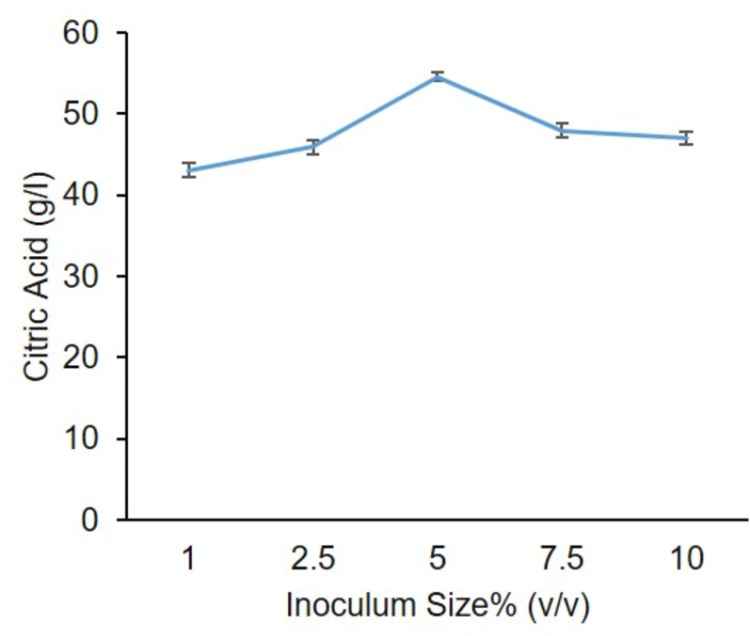
Effect of different inoculum size on citric acid production.

**Fig 7:**
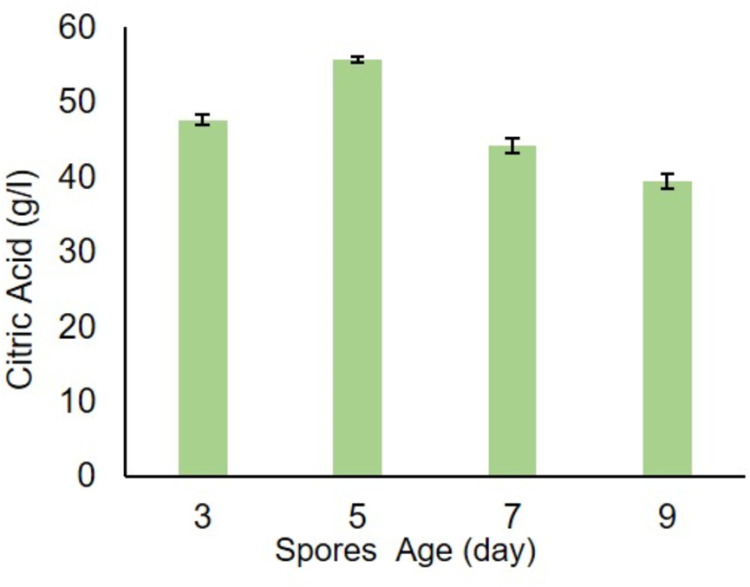
Effect of different spores age (day) on citric acid production.

Different experiments have been performed by varying inoculum size and spore age. The highest citric acid yields 55.0 (g/l) and 56.0 (g/l) were achieved at 5.0% (v/v) inoculum size and 5th-day age spores (Fig 6–7), respectively. Khurshid *et al* [34] also reported comparable findings.

### 6.5. Effect of temperature, pH, and incubation period

**Fig 8:**
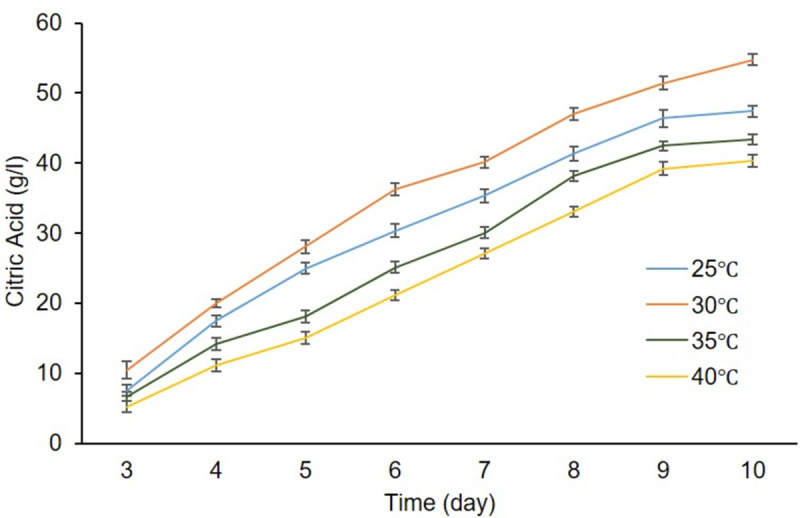
Effect of different incubation temperatures on citric acid production.

**Fig 9:**
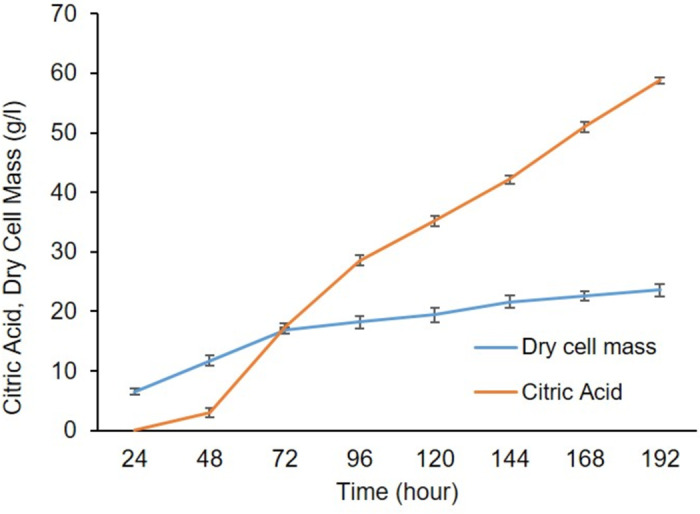
Effect of different incubation time on citric acid production.

The highest (55.0 g/l) and the lowest (47.0 g/l) citric yield were achieved at 30 and 40 C, respectively (Fig 8). After a desired incubation period at 30°C, the highest citric acid production approximately 59.0 g/l was achieved at pH 1.8, dry cell mass of 24.0 g/l and residual sugar was 2.0 g/l as shown in (Fig 9). In the current study, the highest citric acid production was quantified at 30°C, consistent with Shetty [35] findings. Shetty reported that fermentation medium temperature should be between 28–32°C for the highest citric acid production [35]. They achieved the highest citric acid 43.5 g/kg at 32°C using pineapple peel as a carbon source. The optimal temperature analyzed in this study also supports mesophilic nature of *A. niger* which grew well within temperature range of 25–35°C [35]. This temperature range is important for both the optimal growth and production of different industrial products [36,37]. Tim *et al*. [38] also demonstrated that 30°C is the optimal temperature for citric acid production. He explained that citric acid production is affected by reduced cell viability, slow spore germination, denature of different enzymes, and slow metabolic activity at very low or high-value temperatures.

**Fig 10:**
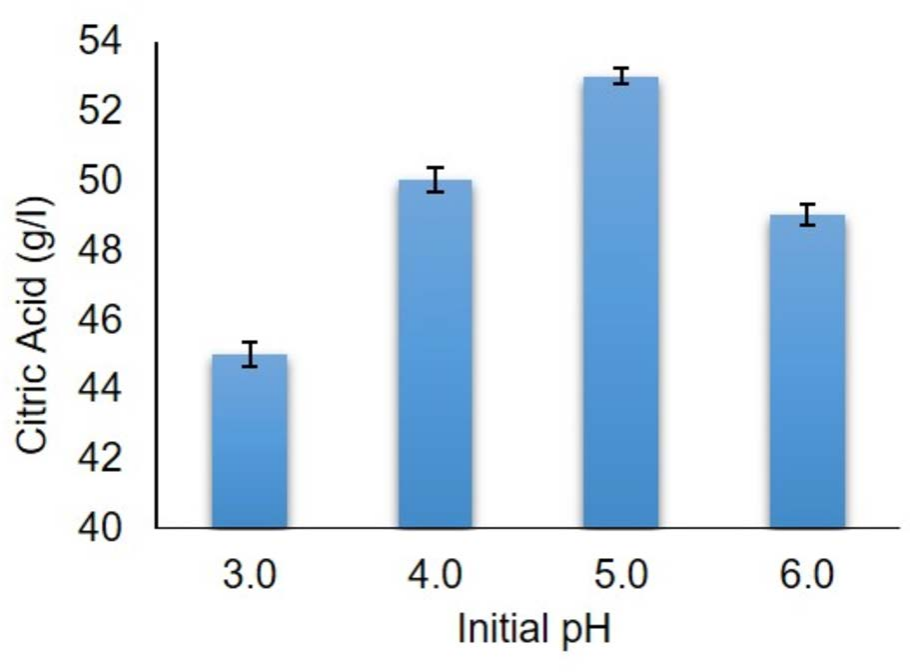
Effect of initial pH on citric acid production.

After a certain incubation period at 30°C, impact of different initial pH on citric acid production by *A. niger* presented the highest citric acid production at pH 5.0 (53.0 g/l). On the other hand, the lowest citric acid yield (45.0 g/l) was analyzed at pH 3.0 (Fig 10). In addition, the maximum citric acid concentration of 53.0 g/l was attained at pH 5.0, aligning with findings from Vidya *et al.,* [39] research as they recorded 31.64 g/l citric acid at pH 5.0 using sucrose along with fruit peel as a carbon source. From the literature, it has been investigated that a pH close to 5.0, plays a crucial role in germination of spores. During germination process, proton ions are released due to the absorption of ammonia, resulting in the release of hydrogen ions. These hydrogen ions lower the pH of medium and favor citric acid production rate [40].

## 7. Toxin analysis

The analytical method was validated using linearity of calibration curve of aflatoxin standards based on their peak areas of AFB1, AFB2, AFG1, and AFG2 respectively in triplicates (Table 2). The calculated regression coefficient was recorded to be R^2^ = 0.9974. After that, the cell free supernatant of citric acid solution, produced by *A. niger* was analyzed through Ultimate dionex 3000 UHPLC. The spectral data was recorded and used for the confirmation and/or quantification of aflatoxins, present in the sample.

**Table 2:** Aflatoxins standards analysis using UHPLC.

Aflatoxin standards	RT (Min)	Peak Area
AFG2	17.472	85241.011
AFG1	20.395	123943.113
AFB2	22.027	175048.498
AFB1	26.147	215582.868

**Fig 11:**
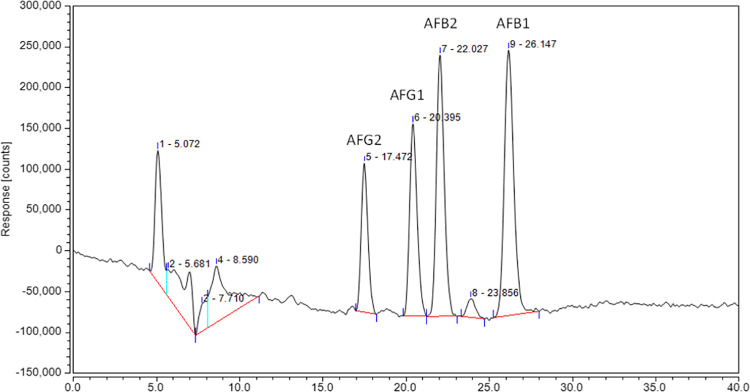
Aflatoxins analysis for standards.

**Fig 12:**
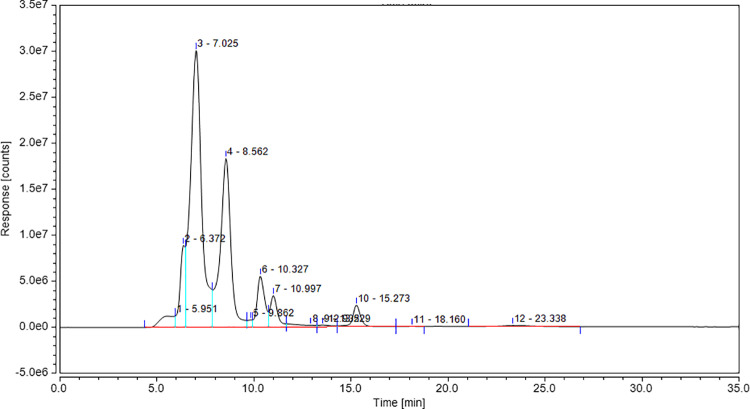
Aflatoxins analysis for cell-free supernatant of citric acid solution, produced by *A. niger.*

The aflatoxins were not detected by comparing the spectral data of aflatoxins standards (Fig 11) with the data obtained during the analysis of a methanolic extract of the cell-free supernatant (Fig 12). This shows the lack of aflatoxins in the citric acid solution, produced by *A. niger* and hence the absence of microbial strains, producing these aflatoxins. Hence, citric acid produced by *A. niger* is safe to use in food industry.

In addition, food-grade nature of *A. niger* with no ability to produce aflatoxin was determined in the current study. Aflatoxins are very toxic and have a potential to cause carcinogenic, mutagenic, and teratogenic effects [41]. UHPLC is an extremely precise and remarkably sensitive technique in analyzing food products and was used to check the presence of aflatoxins in our samples with high accuracy. This advanced technique is useful not only for the quantitative analysis of multiple mycotoxins simultaneously but also for saving the time required for analysis with high accuracy. UHPLC has proved a robust tool specifically designed for mycotoxin analysis in food products [41,42]. Hence, UHPLC analysis has shown that no aflatoxins were produced in fermentation process so citric acid is safe to use in food industry.

## 8. Conclusion

A systematic approach has been used to evaluate different operational parameters to achieve the highest yield of citric acid. The highest yield of citric acid (59.0g/l) was observed at 5.0% inoculum size, initial pH 5.0, and temperature 30°C using sucrose and ammonium nitrate as a carbon and nitrogen source, respectively. Furthermore, evaluation of parameters like media composition, temperature, pH, aeration, and agitation confirms the maintenance of an ideal and better environment for *A. niger* to speed up and efficiently conversion of raw material into citric acid. Hence, aflatoxin-free citric acid can be used in pharmaceutical and food industries as it has been used in bulk amounts in these fields.
